# Antibodies against the Envelope Glycoprotein Promote Infectivity of Immature Dengue Virus Serotype 2

**DOI:** 10.1371/journal.pone.0029957

**Published:** 2012-03-14

**Authors:** Júlia M. da Silva Voorham, Izabela A. Rodenhuis-Zybert, Nilda Vanesa Ayala Nuñez, Tonya M. Colpitts, Heidi van der Ende-Metselaar, Erol Fikrig, Michael S. Diamond, Jan Wilschut, Jolanda M. Smit

**Affiliations:** 1 Department of Medical Microbiology, Molecular Virology Section, University Medical Center Groningen and University of Groningen, Groningen, The Netherlands; 2 Department of Medicine, Section of Infectious Diseases, Medical Institute Yale University School of Medicine, New Haven, Connecticut, United States of America; 3 Department of Molecular Microbiology, and Pathology & Immunology, Washington University School of Medicine, St. Louis, Missouri, United States of America; University of Rochester, United States of America

## Abstract

Cross-reactive dengue virus (DENV) antibodies directed against the envelope (E) and precursor membrane (prM) proteins are believed to contribute to the development of severe dengue disease by facilitating antibody-dependent enhancement of infection. We and others recently demonstrated that anti-prM antibodies render essentially non-infectious immature DENV infectious in Fcγ-receptor-expressing cells. Immature DENV particles are abundantly present in standard (st) virus preparations due to inefficient processing of prM to M during virus maturation. Structural analysis has revealed that the E protein is exposed in immature particles and this prompted us to investigate whether antibodies to E render immature particles infectious. To this end, we analyzed the enhancing properties of 27 anti-E antibodies directed against distinct structural domains. Of these, 23 bound to immature particles, and 15 enhanced infectivity of immature DENV in a furin-dependent manner. The significance of these findings was subsequently tested *in vivo* using the well-established West Nile virus (WNV) mouse model. Remarkably, mice injected with immature WNV opsonized with anti-E mAbs or immune serum produced a lethal infection in a dose-dependent manner, whereas in the absence of antibody immature WNV virions caused no morbidity or mortality. Furthermore, enhancement infection studies with standard (st) DENV preparations opsonized with anti-E mAbs in the presence or absence of furin inhibitor revealed that prM-containing particles present within st virus preparations contribute to antibody-dependent enhancement of infection. Taken together, our results support the notion that antibodies against the structural proteins prM and E both can promote pathogenesis by enhancing infectivity of prM-containing immature and partially mature flavivirus particles.

## Introduction

Dengue virus (DENV) is the leading cause of mosquito-borne viral disease in the world. It is estimated that over 50 million DENV infections occur annually, resulting in 500,000 hospitalizations and over 20,000 deaths [1]. The four antigenically distinct serotypes (DENV 1, 2, 3 and 4) are transmitted to humans by bites of female *Aedes aegypti* and *Aedes albopictus* [1], [2]. Although most infections are asymptomatic, DENV infection may result in a wide spectrum of clinical symptoms, ranging from a febrile illness (dengue fever [DF]) to a life-threatening hemorrhagic and capillary leak syndrome (dengue hemorrhagic fever [DHF]/dengue shock syndrome [DSS]) [1], [2].

The immunopathogenesis of dengue is not fully understood. Infection with one serotype provides lifelong protective immunity to the homologous infecting serotype and cross-protection in the first few months against the other serotypes. However, individuals experiencing a later secondary infection with a distinct DENV serotype are at greater risk for developing severe disease. Additionally, in 6 to 9 month-old children of dengue immune mothers, severe disease is associated with primary infection, possibly because of waning levels of neutralizing maternal antibodies [3], [4]. This latter observation has been the basis for the now widely accepted hypothesis of antibody-dependent enhancement (ADE) of infection as an explanation for the development of DHF/DSS upon infection [5],[6]. Cross-reactive antibodies at sub-neutralizing concentrations are believed to promote virus uptake and infection of Fc-y-receptor-bearing cells, leading to enhanced replication and a more pronounced inflammatory response early in infection [6], [7].

DENV is an enveloped, positive-stranded RNA flavivirus. The virion contains three structural proteins, capsid (C), envelope (E) and membrane (M). In the infected cell, M is formed as a precursor protein called prM. The prM protein acts as a chaperone for correct folding and stabilization of the E protein during virus assembly and egress [8], [9]. E is the primary envelope glycoprotein that mediates the infectious cell entry of flaviviruses [2]. The atomic structure of the E protein ectodomain is organized in three distinct domains [10], [11]. Domain I (DI) is the central domain and participates in the conformational changes required for membrane fusion [12], domain II (DII) contains the highly hydrophobic fusion loop that is inserted into the target cell membrane during the endosomal membrane fusion process [13], [14], [15], and domain III (DIII) has an immunoglobulin-like structure and is believed to be involved in virus attachment to the cell surface [10], [16], [17].

Cells infected with DENV secrete a heterogeneous mixture of structurally distinct virus particles varying from fully immature, partially mature to mature [18], [19], [20], [21]. These virus particles can be distinguished by their difference in size, surface morphology, and the cleavage status of the prM protein [8], [22], [23]. Recent studies found that at least 30–40% of DENV particles released from infected mosquito cells contained prM molecules [21], [24]. The heterogeneity in prM protein expression is caused by inefficient cleavage of prM to M by the host protease furin during virus maturation [25] and can influence the neutralizing and enhancing capacity of antibodies directed against the viral surface proteins [26], [27], [28]. Indeed, anti-prM antibodies render essentially non-infectious immature particles highly infectious [26], [28]. Analogous to virus particle maturation during egress, the internalized antibody-opsonized immature virus particles are processed by cellular furin present in the endocytic pathway, thereby allowing virus infection [26], [29].

The E protein is a major target in the immune response to DENV, and structural analysis demonstrated that some E epitopes are preferentially exposed in immature virions [18]. We recently observed that a weakly neutralizing West Nile virus (WNV) fusion loop antibody has the capacity to render immature WNV particles infectious [30]. The intrinsic ability of E fusion loop antibodies, which are immunodominant in the human humoral response against flaviviruses [31], [32], [33], [34], to render immature particles infectious may therefore pose a threat for the development of a safe and efficacious vaccine against DENV.

In this study, we analyzed the functional properties of a pool of 27 mouse monoclonal antibodies recognizing distinct structural domains to gain a detailed insight in the neutralizing versus enhancing capacity of E antibodies towards immature DENV particles. We found that the majority of antibodies directed against both E DI/II and E DIII can render immature DENV particles infectious in a furin-dependent manner. Furthermore, opsonization of immature WNV with anti-E mAbs and diluted immune serum can cause lethal disease in mice. Thus, in addition to anti-prM antibodies, the vast majority of anti-E antibodies tested can facilitate viral infectivity of immature flavivirus particles, and this may have adverse consequences *in vivo*.

## Results

### Antibodies against the E protein bind to immature dengue virus particles

Initially, we investigated whether anti-E antibodies have the capacity to bind immature DENV particles. Immature DENV-2 strain 16681 particles were produced with an average prM content of 94%±9% in furin-deficient LoVo cells, as described previously [21]. The specific infectivity of the LoVo-derived virus preparation used in this study was 100,000-fold lower compared to that of st virus preparations produced on C6/36 mosquito cells. This is consistent with our earlier results [21], [26] and confirms that immature virus is essentially non-infectious.

The anti-DENV-2 mouse mAb panel used was reported previously and generated after immunization with live DENV-2 strains 16681 and NGC [35]. From this panel, we selected 25 mAbs including 13 that mapped to DIII, 11 that localized to E DI/DII, and 1 that bound E but could not be mapped by yeast surface display of E proteins. The known characteristics of these antibodies are summarized in table 1 (adapted from [35]). Additionally, we tested 2 commercial mAbs, 3H5 (DIII) and 4G2 (DI/DII). All mAbs were tested for binding to immature DENV virions by direct ELISA. We observed that 85% of the E-specific DENV antibodies bound to immature particles (**Table 1**). No consistent difference in binding was seen among mAbs that recognized DI/DII or the DIII domain (43% and 52% positivity, respectively).

**Table 1:** Profile of the antibodies tested.

MAb	Binding domain^a^	Isotype	% Neutralization^b^	Cross-reactivity	Elisa prM DENV^c^	prM-DENV infectivity
DENV2-3H5	E DIII	IgG1	n.d.^d^	none	+	+
DENV2-4G2	E DI, II	IgG2a	n.d.^d^	DENV1,3,4, WNV	+	+
DENV2-29	E DI, II	IgG3	100	DENV1,3,4, WNV	+	+
DENV2-30	E DI, II	IgG2c	100	DENV1,3,4	+	−
DENV2-32	E DI, II	IgG2c	100	none	+	−
DENV2-33	E DI, II	IgG2c	0	DENV3	−	−
DENV2-38	E DIII	IgG3	56	none	+	+
DENV2-40	E DI, II	IgG2b	100	none	+	−
DENV2-44	E DI, II	IgG2c	100	none	+	+
DENV2-46	E DI, II	IgG2c	100	none	+	−
DENV2-48	E DI, II	IgG1	100	none	+	+
DENV2-50	E DI, II	IgG2c	100	none	−	−
DENV2-53	E DI, II	IgG2c	97	DENV1,3	+	+
DENV2-58	E DI, II	IgG2c	100	none	+	+
DENV2-60	E	ND	0	none	+	+
DENV2-67	E DIII	IgG1	92	none	+	−
DENV2-70	E DIII	IgG1	100	none	+	+
DENV2-73	E DIII	IgG2c	100	none	+	+
DENV2-76	E DIII	IgG2c	100	DENV1	+	+
DENV2-77	E DIII	IgG2c	100	DENV1,3,4	+	+
DENV2-93	E DIII	IgG2c	0	none	−	−
DENV2-94	E DIII	IgG2c	93	DENV1	−	−
DENV2-96	E DIII	IgG2c	100	none	+	+
DENV2-97	E DIII	IgG2c	0	none	+	−
DENV2-99	E DIII	IgG2c	75	DENV1	+	−
DENV2-104	E DIII	IgG2c	100	none	+	+
DENV2-106	E DIII	IgG2c	100	none	+	−

a *12 mAbs DI/II; 14 mAbs DIII; 1 unknown.*

b *The percentage of neutralization was determined by single endpoint plaque reduction neutralization assay on BHK21 cells* (adapted from [35]).

c *Positivity indicates at least two times background signal.*

d *n.d. denotes not determined.*

### Anti-E antibodies promote infectivity of immature DENV

**Figure 1:**
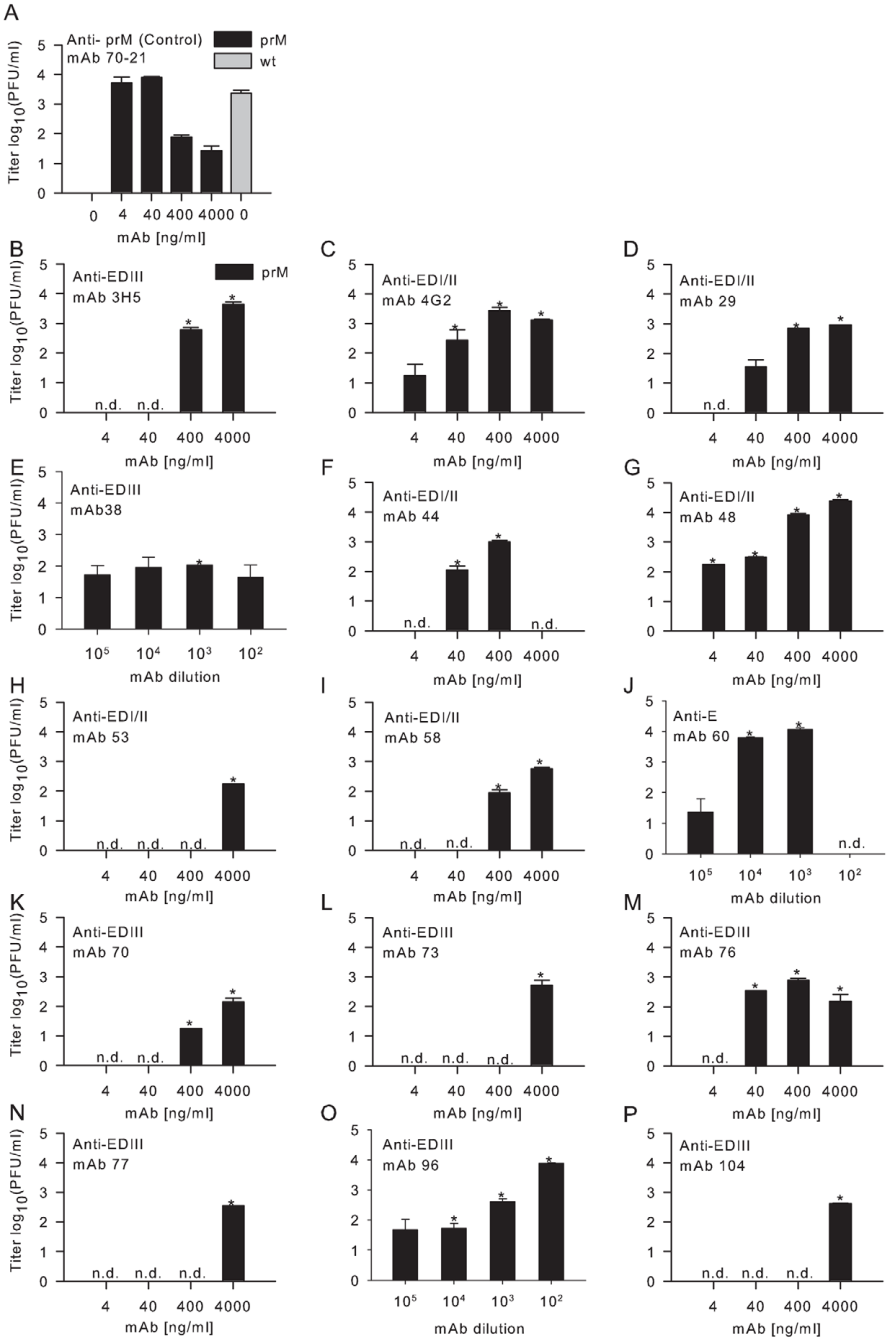
Effect of anti-E mAbs on the infectious properties of fully immature DENV particles. P388D1 cells were infected with immature (prM-containing) DENV-2 strain 16681 at MOG 1000 in the presence or absence of: (**A**) anti-prM antibody and st DENV preparation without antibody, which were used as controls and (**B–P**) different anti-E mAbs. Prior to infection, immature DENV particles were incubated with varying antibody concentrations for 1 h at 37°C. At 43 hpi supernatant was harvested and virus production was analyzed by plaque assay on BHK21-15 cells. Data are expressed as means of at least two independent experiments performed in triplicate. Immature virus in the absence of antibodies was used as a control in all experiments (n = 20), for clarity the average of these results are only plotted in panel 1A. The error bars represent standard deviations (SD); (n.d.) denotes “not detectable”. Student's t-tests were used to determine significance; *, *P*<0.01.

Next, we investigated if the mAbs that bind to immature virus would promote infectivity in murine macrophage-like P388D1 cells, which express three different Fc gamma receptors (FcγRs), FcγRIII [CD16], FcγRII [CD32], and FcγRI [CD64]) [36], [37]. Prior to infection of P388D1 cells, immature DENV was pre-incubated for 1 hr at 37°C in the presence or absence of increasing concentrations of anti-prM or anti-E antibodies and added to P388D1 at a multiplicity of 1000 genome-containing particles (GCP) per cell (MOG 1000) as determined by quantitative PCR (qPCR) analysis. At 43 hr post-infection (hpi), the supernatant was harvested, and infectious virus production was analyzed by plaque assay on BHK21-15 cells. Consistent with previous studies [26], [28], immature DENV particles became infectious in the presence of anti-prM with titers comparable to that of st virus preparations in the absence of antibody (**Fig. 1A**). Of the 23 E mAbs tested, 15 mAbs (65%) facilitated infectivity of immature DENV particles (Table 1). However, different patterns of enhancement were observed. MAbs 4G2 (DI/II), DV2-29 (DI/II), DV2-48 (DI/II), DV2-60 (E), DV2-76 (DIII), and DV2-96 (DIII) (**Fig. 1C, D, G, J, M, and O**, respectively) promoted infectivity of immature DENV over a broad antibody concentration range and to levels comparable of infection of st DENV particles in the absence of antibodies. In comparison, DV2-38 (DIII) (**Fig. 1E**) enhanced viral infectivity at all concentrations tested, albeit with lower efficiency. MAbs 3H5 (DIII), DV2-44 (DI/II), DV2-53 (DI/II), DV2-58 (DI/II), DV2-70 (DIII), DV2-73 (DIII), DV2-77 (DIII) and DV2-104 (DIII) (**Fig. 1B, F, H, I, K, L, N, and P**, respectively) modestly promoted viral infectivity of immature DENV, and only at higher antibody concentrations. Thus, anti-E DI/II and anti-E DIII antibodies have the capacity to rescue the infectivity of immature DENV particles. Notably, whereas enhancement of infection of immature DENV particles opsonized with anti-prM antibodies was more readily seen at lower antibody concentrations (**Fig. 1A**) and [26], [28], enhancement of infection mediated by most anti-E antibodies was preferentially observed at higher antibody concentrations. Interestingly, six mAbs that mapped to DIII and six mAbs that localize to DI/DII (**Table 1**) failed to promote viral infectivity of immature particles in P388D1 cells at any antibody concentration tested. These mAbs also failed to promote viral infectivity of immature particles in human U937 cells (data not shown).

### Anti-E mAbs facilitate binding of immature DENV particles to cells

**Figure 2:**
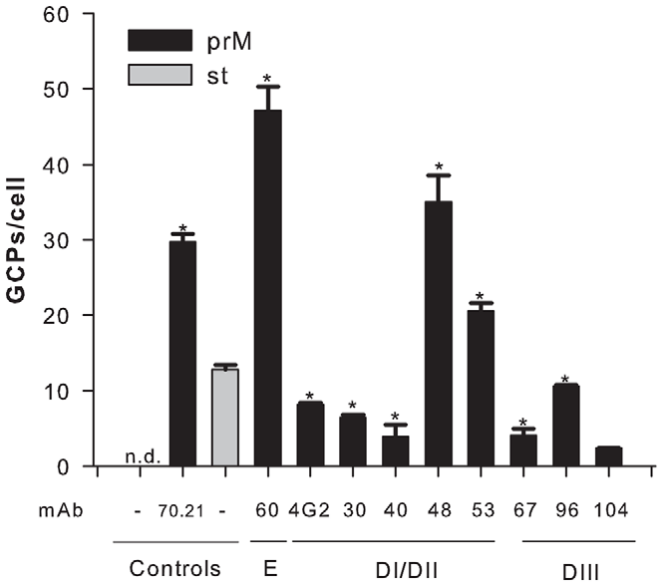
Antibodies facilitate binding and internalization of fully immature DENV particles. Binding of immature and standard virion preparations to P388D1 cells with and without prior virus opsonization with antibodies. Virus-cell binding/internalization was measured after 1 h incubation at 37°C by qPCR analysis. Results are shown at conditions of efficient ADE for mAbs that promote viral infectivity. For mAbs that neutralize viral infectivity, a wide antibody concentration range was tested and the condition at which the highest number of GCPs bound per cell is observed is depicted. Data are expressed as means of two independent experiments performed in triplicate. The error bars represent standard deviations (SD); (n.d.) denotes “not detectable”. Student's t-tests were used to determine significance; *, *P*<0.01.

To investigate if mAbs directed against E, analogous to that seen with enhancing anti-prM antibodies [26], promote uptake of immature DENV particles in cells, we performed a cell binding and internalization assay using qRT-PCR [38]. For this purpose, we selected a subset of enhancing and neutralizing anti-DENV-2 mAbs directed against distinct domains of the E protein. DENV-immune complexes were added to P388D1 cells at a MOG of 1000 and incubated for 1 hr at 37°C. All tested anti-E antibodies facilitated cell binding and internalization of immature particles to levels (3- to 47-fold, *P<0.01) greater than those observed for immature particles in the absence of antibody (**Fig. 2**). Antibody-opsonized immature particles appeared to bind to cells as efficiently as or even slightly better (mAbs DV2-60, DV2-48 and DV2-53, P<0.01) than st DENV in the absence of antibody. The neutralizing mAbs DV2-30, DV2-40 and DV2-67 bound to a somewhat lower extent to cells than st DENV particles, albeit to comparable levels as the enhancing anti-E mAbs 4G2, DV2-96 and DV2-104. This suggests that neutralization of infection by these mAbs likely occurs in part, at a post-attachment stage after binding of the virus-immune complexes to cells.

### Enhancement of immature DENV infectivity by anti-E mAbs is furin-dependent

**Figure 3:**
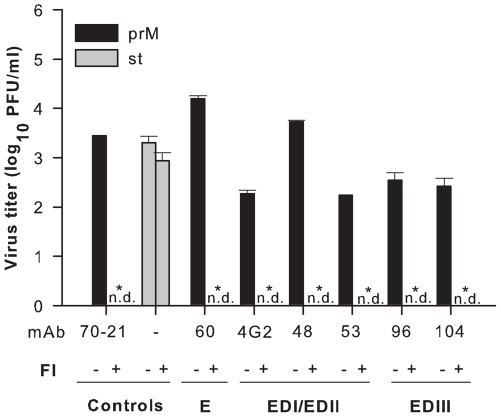
Antibody dependent enhancement is critically dependent on furin activity. P388D1 cells were infected with st DENV or antibody-opsonized immature particles in the presence of absence of furin inhibitor. Data are expressed as means of three independent experiments performed in duplicate. The error bars represent standard deviations (SD); (n.d.) denotes “not detectable”. Student's t-tests were used to determine significance; *, *P*<0.01.

The enzymatic activity of furin is crucial for rendering immature DENV particles infectious after opsonization with anti-prM mAbs [26]. As such, we investigated whether furin activity also was required for the infectivity of immature particles opsonized with anti-E mAbs by treating P388D1 cells with furin inhibitor (FI), decanoyl-L-arginyl-L-valyl-L-lysyl-L-arginyl-chloromethylketone (decRRVKR-CMK) prior to and during infection. The half-life of FI in cells is approximately 4–8 hrs [39]. This was confirmed in a control experiment in which P388D1 cells were infected with st DENV particles with and without FI treatment. Notably, addition of FI to P388D1 cells did not reduce infectivity, indicating that for st DENV FI is no longer active when progeny virions are released from the infected cell (**Fig. 3**). Instead, repeated addition of FI to the producer cell at the time of virion assembly markedly reduced the formation of infectious particles implying that under these conditions only non-infectious immature particles are being released (data not shown and [26]). The latter control confirms that FI actively interferes with furin activity. Notably, inhibition of furin activity in target cells abolished infectivity of antibody-opsonized immature virions, demonstrating that furin activity during the entry stage is crucial for rendering DENV-immune complexes infectious (**Fig. 3**).

### Anti-E-mediated enhancement of infectivity of prM-containing particles from standard DENV preparations

**Figure 4:**
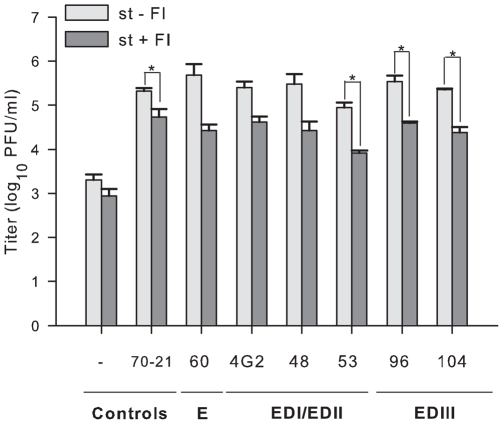
Effect of furin inhibitor on the infectivity of standard virus preparations. P388D1 cells were infected with antibody-opsonized st DENV particles in the presence or absence of furin inhibitor. Standard DENV particles without antibody or opsonized with anti-prM antibody 70.21 were used as controls. The error bars represent standard deviations (SD) derived from at least two separate experiments performed in duplicate. Two-tailed Student's t-tests were used to determine significance; *, *P*<0.01.

Multiple studies have shown that anti-E mAbs facilitate ADE of st DENV preparations [40], [41], [42]. Here, we show that anti-E mAbs promote infectivity of immature virions. As the accessibility of an epitope on a particular virion structure could impact the ability of anti-E mAbs to enhance infectivity, we next investigated to what extent furin activity was required for ADE of st DENV preparations after opsonization with E antibodies. Initially, the viral infectivity of st DENV preparations in P388D1 cells was analyzed in the presence of increasing mAb concentrations. As expected, all mAbs tested enhanced infectivity of st DENV (**Fig. S1**). Enhancement of infection was seen over a broad range of antibody concentrations: under optimal ADE conditions an approximately 100-fold increase in virus particle production was observed. Next, the FI assay was performed at the single mAb concentration that most efficiently promoted ADE: for the E mAbs 4G2, DV2-48, and DV2-53 a concentration of 400 ng/ml, for DV2-104 a concentration of 4000 ng/ml, and for DV2-60 and DV2-96 a 1/10^3^ dilution of hybridoma supernatant was used. Notably, furin activity was necessary for optimal ADE of st DENV opsonized with anti-E mAbs, as an approximate ∼10-fold reduction in viral infectivity was observed in cells treated with FI (**Fig. 4**). Thus, a fraction of the ADE observed with st DENV preparations is caused by infectivity of fully or partially immature particles that required furin cleavage.

### Immature WNV particles opsonized with anti-E 4G2 induce lethal disease in mice

**Figure 5:**
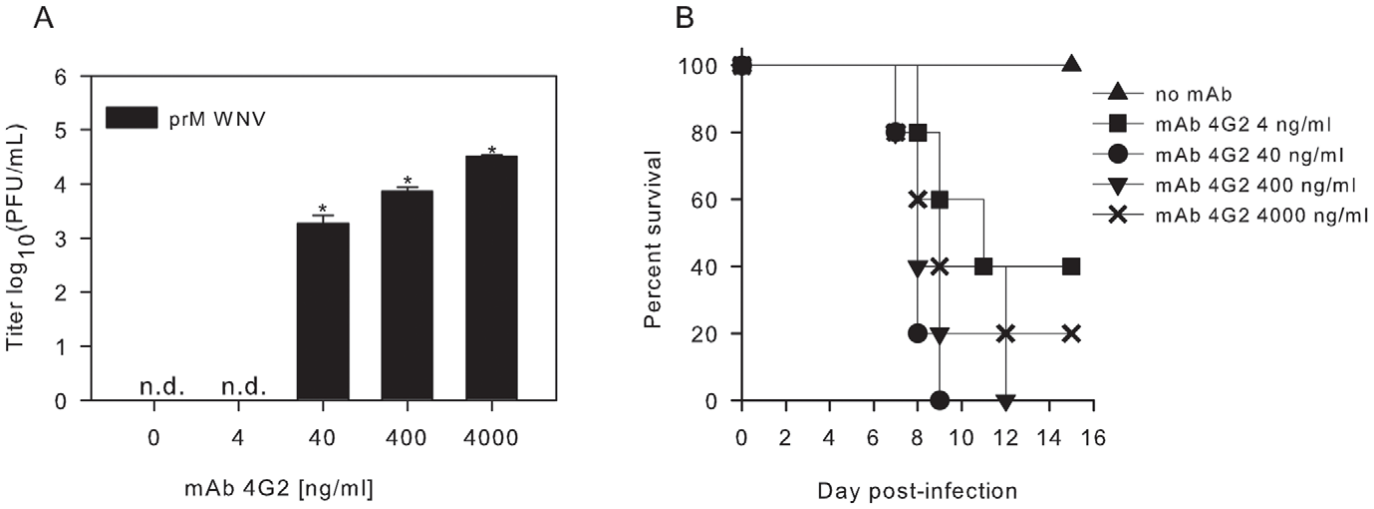
Effect of anti-E mAb 4G2 on the infectious properties of immature WNV particles *in vitro* and *in vivo*. (**A**) P388D1 cells were infected with immature WNV opsonized with increasing concentrations of 4G2 at MOG 10. At 26 hpi, the supernatant was harvested and virus production was analyzed by plaque assay on BHK21-15 cells. Data are expressed as means of at least two independent experiments performed in duplicate. The error bars represent standard deviations (SD); (n.d.) denotes “not detectable”. Student's t-tests were used to determine significance; *, *P*<0.01. (**B**) Immature WNV was incubated with different concentrations of anti-E 4G2 for 1 hr at 37°C, and injected in mice. A total of 3.4×10^7^ GCPs were given per mouse. Five mice were used for each experimental condition.

Because wild type mice do not support DENV replication, and thus are not a suitable animal model for pathogenesis, we instead tested the significance of our findings using the well-established C57BL/6 mouse model of WNV infection [43]. Immature WNV was produced on LoVo cells, as described before [21], [44]. Consistent with our published results, the specific infectivity of the immature WNV preparation was reduced ∼30,000-fold compared to that of st virus prepared in BHK21 cells. Next, we evaluated the infectious properties of immature WNV opsonized with the flavivirus cross-reactive anti-E mAb 4G2 in P388D1 cells at MOG 10. At 26 hpi, the supernatant was harvested and virus production was analyzed by plaque assay on BHK21-15 cells. In agreement with our data obtained for DENV (**Fig. 1C**), 4G2 promoted viral infectivity of immature WNV (**Fig. 5A**). Subsequently, mice were inoculated by intraperitoneal (IP) injection with immature WNV particles with and without prior opsonization with 4G2. Five mice were used for each experimental condition, and 3.4×10^7^ GCPs were inoculated per mouse (based on 10^4^ infectious units for st virus preparations). Mice injected with immature WNV in the absence of Abs showed no clinical signs of infection or lethality, confirming that immature WNV by itself is not infectious (**Fig. 5B**). In comparison, immature WNV opsonized with anti-E mAb 4G2 became infectious in mice. At a mAb concentration of 4 ng/ml 3 out of 5 mice died, at 40 and 400 ng/ml all mice died and at a 4G2 concentration of 4000 ng/ml 4 out of 5 mice died (**Fig. 5B**). These results demonstrate that an anti-E mAb directed against DI/II can render immature WNV infectious *in vivo* in a dose-dependent manner.

### Immune serum induces ADE of immature WNV

**Figure 6:**
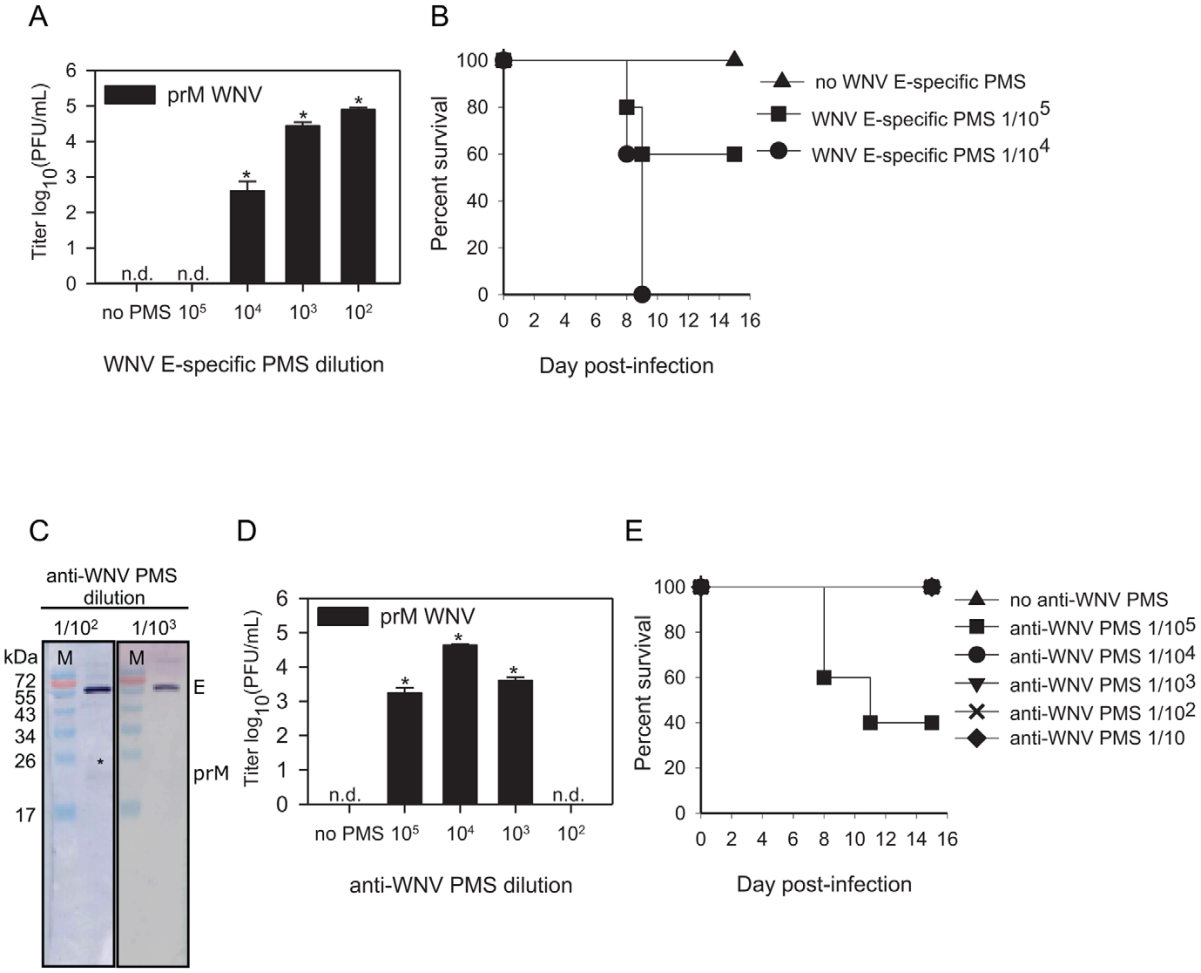
Effect of immune sera on the infectious properties of immature WNV particles. Infectivity and mice experiments were performed as described in the legend to Fig. 5. (**A**, **B**) immune sera from mice prior vaccinated with E ectodomain. (**D**, **E**) Immune serum derived from mice prior infected with a sublethal dose of st WNV. (**A**, **D**) Values depicted on the x axis represent dilution factors. The error bars represent standard deviations (SD); (n.d.) denotes “not detectable”, (PMS) denotes polyclonal mouse serum. Student's t-tests were used to determine significance; *, *P*<0.01. (**C**) Western blot analysis of immune serum from mice prior infected with a sublethal dose of WNV. On the SDS-PAGE gel, purified immature WNV was loaded.

The ability of anti-E Abs to render immature particles infectious could have implications for the development of a safe and efficacious tetravalent DENV vaccine as all vaccine formulations in development aim to induce a robust polyclonal response to E. To test the intrinsic ability of polyclonal anti-E Abs to rescue the infectivity of immature WNV particles, we analyzed the infectious properties of immature particles in presence of immune serum derived from mice vaccinated with E ectodomain. Serum from mice immunized with the ectodomain of E rendered immature WNV infectious in P388D1 cells at a broad antibody concentration range (1/10^2^ to 1/10^4^ dilution) (**Fig. 6A**). Furthermore, mice injected IP with immature WNV (3.4×10^7^ GCPs/mice) pre-opsonized with anti-E ectodomain sera succumbed to lethal infection in a dose-dependent manner (**Fig. 6B**). At an anti-E ectodomain serum dilution of 1/10^4^ all mice died whereas at a 1/10^5^ serum dilution 2 out of 5 mice succumbed to WNV infection. Thus, polyclonal antibodies generated in mice upon vaccination with E ectodomain can render immature virions infectious. These intriguing results prompted us to evaluate the enhancing properties of serum derived from mice infected with a sublethal dose of WNV. First, the serum was subjected to Western blot analysis to determine the ratio of anti-prM and anti-E antibodies. Notably and consistent with prior studies [34], [45], the vast majority of anti-virion Abs present in WNV immune serum were directed against the viral E protein (**Fig. 6C**). Thereafter, we analyzed the infectious properties of immature WNV particles opsonized with increasing concentrations of immune serum (1/10 to 1/10^8^ dilution) in P388D1 cells at MOG 10. Enhancement of immature WNV infectivity was observed at a serum dilution of 1/10^3^ to 1/10^5^, with titers 3 to 4 logs higher than those obtained with immature virus in the absence of antibody (**Fig. 6D**). Subsequent *in vivo* experiments revealed that all mice receiving immune serum at dilutions of 1/10 to 1/10^4^ survived infection, whereas 3 out of 5 animals inoculated with immature WNV opsonized with serum at a dilution of 1/10^5^ succumbed to lethal infection (**Fig. 6E**).

## Discussion

In this study, we demonstrated that, in addition to anti-prM antibodies [26], [28], anti-E antibodies can promote infectivity of immature DENV by facilitating internalization and maturation of immature DENV particles in FcyR-expressing cells. Accordingly, and in agreement with previous data with anti-prM antibodies [26], we found that enzymatic activity of furin in the target cell was required for facilitating infectivity of anti-E antibody-opsonized immature particles. The significance of this finding was confirmed with WNV, as low concentrations of immune serum promoted infectivity of immature WNV particles in vitro and in vivo. Furthermore, detailed investigation of the enhancing properties of anti-E antibodies in st DENV preparations revealed that enhancement of infection also is promoted by furin activity present within the target cell. These results demonstrate that anti-E antibodies can render immature flavivirus particles infectious and that enhancement of infection is modulated by the maturation status of the virus.

The majority of the anti-E mAbs tested in this study bound to immature DENV particles. While some DI/II- and DIII-specific anti-E antibodies promoted infection, others did not. In both situations, the antibodies facilitate binding and uptake of immature virions into an endocytic or phagocytic pathway of the target cell. For those mAbs promoting productive infection, we postulate that the low-pH environment in endosomes induces a structural transition change in the virion that allows furin to cleave prM to M allowing membrane fusion and infection. Anti-E mAbs that do not stimulate viral infectivity may interfere with this conformational change of the virion prior to furin cleavage, or with the fusion process itself. Indeed, previous analysis of an anti-E WNV fusion loop antibody revealed that the fusion loop mAb E53 stabilizes the viral spike complex of immature DENV particles to such an extent that a lower pH environment is required to trigger the structural transition change of the virion [30]. In other words, the fate of the immature DENV-immune complex is determined in the endocytic/phagocytic pathway of the cell.

A distinct enhancement pattern was observed for ADE of immature virus opsonized with anti-E mAbs compared to that of anti-prM mAbs. ADE of anti-E opsonized immature particles only was observed at high antibody concentration, whereas for anti-prM mAbs ADE was seen at lower rather than higher concentrations. One possible explanation for this is that fully immature particles have relatively few accessible epitopes available for engagement by our panel of anti-E mAbs. A smaller number of available epitopes might require a higher fractional occupancy and thus higher concentrations, to reach a stoichiometry sufficient for enhancement. Indeed, structural data confirm that in immature flaviviruses, the E protein is largely covered by the prM protein, which could limit epitope exposure of some E protein epitopes [12], [20], [46], [47]. Consistent with this hypothesis, several of our mAbs (DV2-29, DV2-44, DV2-48, DV2-58, DV2-70, DV2-73, DV2-76, DV2-77, DV2-77, DV2-96 and DV2-104) were strongly neutralizing against st DENV preparations [35] but showed little inhibitory activity of opsonized immature particles even at concentrations that were 10–100-fold above their EC50. While engagement of a relatively small number of accessible E protein epitopes was sufficient for enhancement of st WNV preparations on CD32-expressing K562 cells [42] the precise stoichiometry of enhancement for immature virions or other cell types that express distinct sets of FcγR remains to be determined.

Coupled with prior studies, our observation that both anti-prM and anti-E antibodies can promote infectivity of immature DENV particles supports the hypothesis that immature DENV particles are important in disease pathogenesis [26], [28]. Whereas most prM antibodies are poorly neutralizing and thus, efficiently enhance infection of partially mature and immature particles, anti-E antibodies, depending on the concentration and epitope accessibility can neutralize or enhance infection of immature particles. During homologous re-infection with the same serotype, the anti-E antibodies may more readily neutralize infection whereas during heterologous re-infection with a different serotype cross-reactive anti-prM and anti-E antibodies may bind yet not neutralize, setting the stage for ADE. The importance of virion maturation in controlling viral infectivity during natural infection remains unclear. While it is unknown what state of maturation DENV particles exists *in vivo*, prM antibodies appear frequently in the context of the human humoral response [28]. Our in vivo data with WNV, suggests that immature virions opsonized with serum Abs can promote enhanced infection and disease. As such, evaluation of the enhancing and neutralizing properties of anti-DENV serum with virions with distinct maturation states may help to define better correlates of protection or pathogenesis during secondary heterosubtypic infection.

The observation that the maturation state of a virus preparation influences the neutralizing or enhancing potential of an antibody has implications for DENV vaccine development. This report clearly shows that antibodies generated upon vaccination with E ectodomain can facilitate infectivity of immature WNV particles. Also, low concentrations of immune serum from mice prior infected with a sublethal dose of WNV stimulated viral infectivity of immature virions. However, our analysis also shows that a number of antibodies recognizing E DI/II and DIII epitopes exist that do not promote the infectivity of immature particles. Future detailed examination of these antibodies may define novel targets for vaccine refinement. In general, immunization or boosting strategies that minimize induction of anti-prM or cross-reactive anti-E antibodies may limit enhancement of infection of partially mature and immature particles during subsequent natural challenge.

## Materials and Methods

### Ethics statement

This study was carried out in strict accordance with the recommendations Guide for the Care and Use of Laboratory Animals of the National Institutes of Health. All animal experimental protocols were approved by the Institutional Animal Care and Use Committee of Yale University (Protocol Permit Number: 2008-07941) and experiments were done in a Biosafety Level 3 animal facility according to the regulations of Yale University. All efforts were made to minimize suffering.

### Cells

*Aedes albopictus* C6/36 cells were maintained in minimal essential medium (Life Technologies) supplemented with 10% fetal bovine serum (FBS), 25 mM HEPES, 7.5% sodium bicarbonate, penicillin (100 U/ml), streptomycin (100 µg/ml), 200 mM glutamine and 100 µM nonessential amino acids at 30°C, 5% CO_2_. Baby hamster Kidney (BHK21) and BHK21 clone 15 cells (BHK21-15) cells were cultured in DMEM (Life Technologies) containing 10% FBS, penicillin (100 U/ml), streptomycin (100 µg/ml), 10 mM HEPES, and 200 mM glutamine. Human adenocarcinoma LoVo cells were cultured in Ham's medium (Invitrogen) supplemented with 20% FBS at 37°C, 5% CO_2_. Mouse macrophage P388D1 cells were maintained in DMEM supplemented with 10% FBS, penicillin (100 U/ml), and streptomycin (100 µg/ml), sodium bicarbonate (Invitrogen, 7,5% solution) and 1.0 mM sodium pyruvate (GIBCO) at 37°C, 5% CO_2_.

### Virus growth

DENV-2 strain 16681 and WNV strain NY385-99 were propagated on C6/36 cells and BHK21 cells respectively, as described before [21], [38]. Immature DENV and WNV particles were produced on LoVo cells as described previously [21]. Briefly, LoVo cells were infected at MOI 5 for DENV and MOI 4 for WNV. Virus inoculum was removed after 1.5 hr and fresh medium was added after washing the cells three times with PBS. At 72 hpi, the medium containing the virus particles was harvested, cleared from cellular debris by low-speed centrifugation, aliquoted, and stored at −80°C. The specific infectivity of the DENV and WNV preparations was determined by measuring the number of infectious units by plaque assay on BHK21-15 cells and the number of GCPs by quantitative PCR (qPCR) analysis, as described previously [21], [38].

### qPCR

To determine the number of GCP, we extracted viral RNA by use of a QIAamp viral RNA mini kit (QIAGEN, Venlo, The Netherlands). cDNA was synthesized from viral RNA by RT-PCR. For DENV we used a published protocol [38]. For WNV, the forward primer 5′-GTT GGC GGC TGT TTT CTT TC-3′, and the reverse primer 5′-GGG ATC TCC CAG AGC AGA ATT-3′ and a TaqMan probe 5′-FAM-AAT GGC TTA TCA CGA TGC CCG CC-TAMRA-3′ (Eurogentec, Seraing, Belgium) was used. DNA was amplified for 40 cycles (15 s at 95°C and 60 s at 60°C) on a StepOne Real-Time PCR instrument (Applied Biosystems, Carlsbad, CA) and the concentration GCPs was determined using a standard curve based on a cDNA plasmid encoding the nonstructural genes of WNV NY99 (kind gift from Dr. G.P. Pijlman, Wageningen University, The Netherlands).

### ELISA

The binding properties of anti-E antibodies to immature virus particles were assessed with a three-layer ELISA. Briefly, microtiter ELISA plates (Greiner bio-one) were coated with 5×10^8^ GCP of purified virus preparations per well in 100 µl coating buffer, overnight. After blocking with 2% milk in coating buffer for 120 min, 100 µl of two-fold serial dilutions of anti-E DENV mAbs were applied to the wells and incubated for 1.5 hr, in triplicate. Subsequently, 100 µl of horseradish peroxidase-conjugated goat anti-mouse IgG isotype antibody (Southern Biotech) was applied for 1 hr. All incubations were performed at 37°C. Staining was performed using o-phenylene-diamine (OPD) (Eastman Kodak Company) and absorbance was read at 492 nm (A_492_) with an ELISA reader (Bio-tek Instruments, Inc.). Samples were considered positive when the absorbance was two times higher than the background signal.

### Infectivity assays

Virus or virus-antibody complexes were added to a monolayer (2×10^5^) of P388D1 cells in 24 wells plates (Costar), at a multiplicity of genome-containing particles (MOG) per cell of 1000 for DENV and 10 for WNV. After 1.5 h incubation at 37°C, fresh medium was added to the cells. At 26 hpi for WNV and 43 hpi for DENV, the medium was harvested and virus production was analyzed by plaque assay on BHK21-15 cells, as described previously [48]. The limit of detection in the plaque assay is 20 PFU/ml. In furin inhibitor experiments, cells were treated with 25 µM of furin-specific inhibitor, decanoyl-L-arginyl-L-valyl-L-lysyl-L-arginyl-chloromethylketone (decRRVKR-CMK) (Calbiochem) prior to and during virus infection [26].

### Binding assays

To determine the number of bound and/or internalized genome-containing particles per cell, virus or virus-antibody complexes were incubated with 2×10^5^ P388D1 cells at MOG 1000 for 1 h at 37°C. Subsequently, cells were washed three times with ice-cold PBS containing MgCl_2_ and CaCl_2_ (Life Technologies) to remove unbound virus-antibody complexes. Viral RNA was extracted subsequently from the cells using the QIAamp Viral RNA mini Kit (QIAGEN). Thereafter, cDNA was synthesized from the viral RNA with reverse transcription-PCR (RT-PCR), and the copy number was quantified using qPCR [38]. The limit of detection is 1.6 GCPs bound per cell.

### Immune sera and Western Blot analysis

Immune WNV serum was produced by injecting mice IP with a sublethal dose of a st WNV preparation. At day 28, blood was collected by orbital puncture. Plasma was obtained, aliquoted, and stored at 4°C. Anti-E immune sera was obtained in the same manner from mice injected IP with the E ectodomain. Both sera were tested via Western blot analysis for reactivity to E protein as well as for ability to neutralize wt WNV *in vitro*. For Western blot analysis, 3.0×10^9^ GCP of purified immature WNV was loaded on 12.5% SDS polyacryramide gels under non-reducing conditions and electroblotted in the presence of two concentrations of immune serum, 1∶1000 and 1∶100.

### Mouse experiments

All animal experimental protocols were approved by the Institutional Animal Care and Use Committee of Yale University and experiments were done in a Biosafety Level 3 animal facility according to the regulations of Yale University. Female C57BL/6 mice (6 weeks old) were infected by IP injection with immature WNV prior opsonized with increasing concentrations anti-E mAb 4G2 or immune serum. Five mice were used for each experimental condition, and 3.4×10^7^ GCPs were inoculated per mouse and mice were followed for 15 days.

## Supporting Information

Figure S1 **Effect of mAbs on the infectious properties of standard DENV preparations.** (**A–G**) P388D1 cells were infected with DENV-2 strain 16681 at MOG 1000 in the presence or absence of different anti-E mAbs. At 43 hpi supernatant was harvested and virus production was analyzed by plaque assay on BHK21-15 cells. Data are expressed as means of at least two independent experiments. St virus in the absence of antibodies was used as a control in all experiments (n = 6), for clarity the average of these results are only plotted in panel A. The error bars represent standard deviations (SD). Two-tailed Student's t-tests were used to determine significance; *, *P*<0.01.
(EPS)
